# Development of a thick and functional human adipose-derived stem cell tissue sheet for myocardial infarction repair in rat hearts

**DOI:** 10.1186/s13287-023-03560-9

**Published:** 2023-12-20

**Authors:** Jingbo Zhang, Junjun Li, Xiang Qu, Yuting Liu, Akima Harada, Ying Hua, Noriko Yoshida, Masako Ishida, Akiko Tabata, Lifu Sun, Li Liu, Shigeru Miyagawa

**Affiliations:** 1 Department of Cardiovascular Surgery, Osaka University Graduate School of Medicine, 2-2 Yamada-Oka, Suita, Osaka 565-0871 Japan; 2 Frontier of Regenerative Medicine, Osaka University Graduate School of Medicine, 2-2 Yamada-Oka, Suita, Osaka 565-0871 Japan

**Keywords:** Adipose-derived mesenchymal stem cell, Tissue engineering, Regenerative medicine, Myocardial infarction, Angiogenesis

## Abstract

**Background:**

Heart failure (HF) is a major cause of death worldwide. The most effective treatment for HF is heart transplantation, but its use is limited by the scarcity of donor hearts. Recently, stem cell-based therapy has emerged as a promising approach for treating myocardial infarction. Our research group has been investigating the use of human induced pluripotent stem cell-derived cardiomyocyte patches as a potential therapeutic candidate. We have successfully conducted eight cases of clinical trials and demonstrated the safety and effectiveness of this approach. However, further advancements are necessary to overcome immune rejection and enhance therapeutic efficacy. In this study, we propose a novel and efficient technique for constructing mesenchymal stem cell (MSC) tissue sheets, which can be transplanted effectively for treating myocardial infarction repair.

**Methods:**

We applied a one-step method to construct the human adipose-derived mesenchymal stem cell (hADSC) tissue sheet on a poly(lactic-co-glycolic acid) fiber scaffold. Histology, immunofluorescence, and paracrine profile assessment were used to determine the organization and function of the hADSC tissue sheet. Echocardiography and pathological analyses of heart sections were performed to evaluate cardiac function, fibrosis area, angiogenesis, and left ventricular remodeling.

**Results:**

In vitro, the hADSC tissue sheet showed great organization, abundant ECM expression, and increased paracrine secretion than single cells. In vivo, the hADSC tissue sheet group demonstrated improved cardiac functional recovery, less ventricular remodeling, decreased fibrosis, and enhanced angiogenesis than the MI group.

**Conclusions:**

We developed thick and functional hADSC tissue sheets via the one-step strategy. The hADSC tissue sheet showed excellent performance in treating myocardial infarction in the rat model.

**Graphical Abstract:**

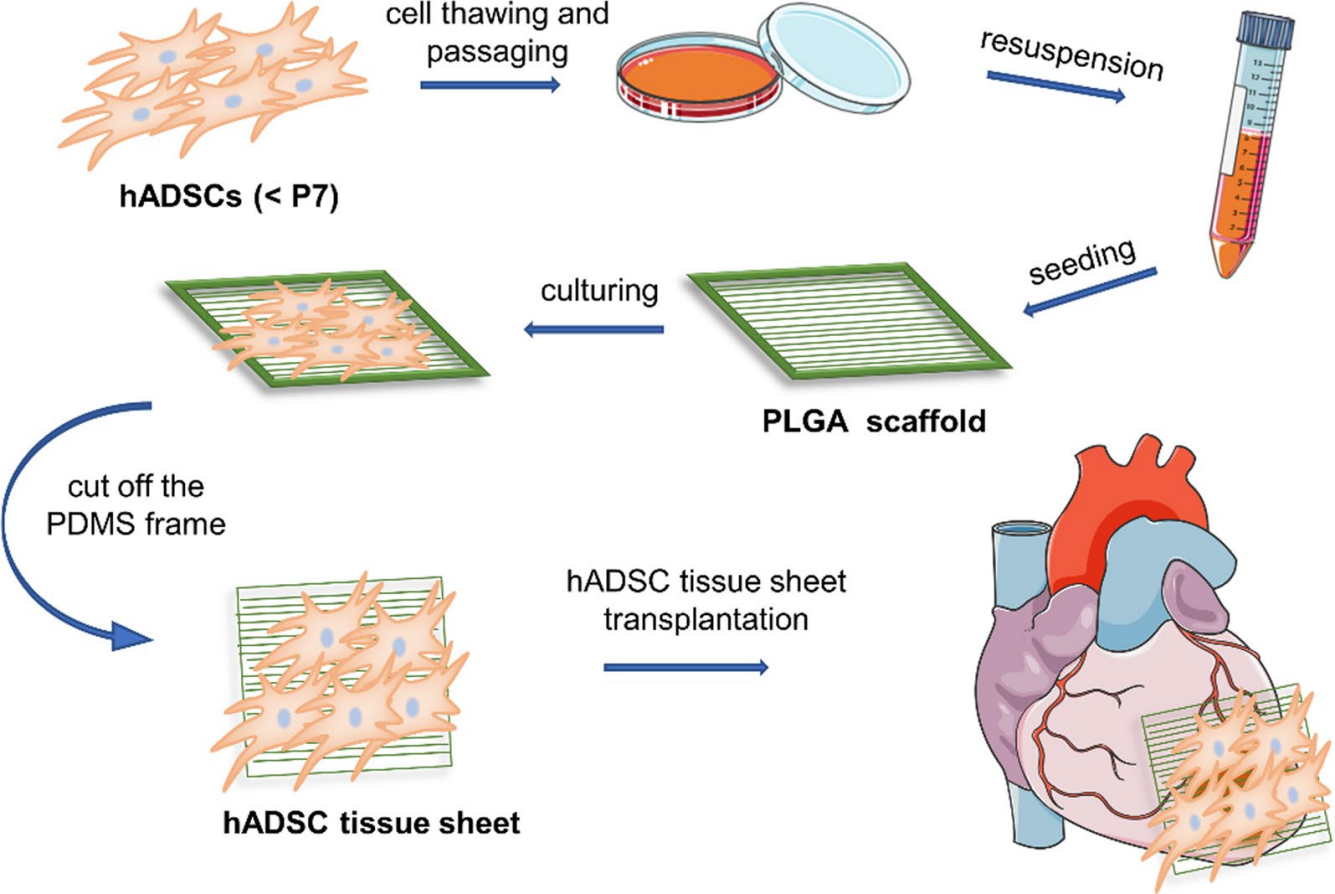

**Supplementary Information:**

The online version contains supplementary material available at 10.1186/s13287-023-03560-9.

## Introduction

Ischemic heart disease is a leading cause of morbidity and mortality [1, 2], with myocardial infarction (MI) being the most common cause of heart failure (HF) worldwide [3]. MI results in extensive cardiomyocyte death, leading to a critically intense inflammatory reaction and the creation of a second lesion in the heart [4]. Although clinical treatments for MI can achieve myocardial reperfusion, cardiomyocyte death caused by ischemia cannot be reversed [5]. The loss of cardiomyocytes and the development of noncontractile scar tissue lead to heart deterioration and impaired function. Cardiac regeneration in adulthood is restricted, and the ultimate restoration of cardiac function can only be achieved via artificial hearts or heart transplantation. However, artificial heart transplantation is associated with high medical costs, postoperative bleeding, infections, and other complications, while allogeneic heart transplantation faces constraints such as a shortage of sources, endemic infections, and high medical expenses [6].

Cell-based therapies have emerged as prominent and novel treatment options for myocardial regeneration. Among them, mesenchymal stem cells (MSCs) have emerged as a leading approach in the field of regenerative medicine for cardiovascular diseases. MSCs offer several advantages over other types of stem cells, making them widely used in both basic research and clinical studies. These advantages include easy acquisition, low immunogenicity, rapid proliferation, and the absence of ethical concerns [7, 8]. Adipose-derived MSCs (ADSCs) have been shown to improve cardiac contractility and remodeling with angiogenesis via the secretion of several cytokines, including hepatocyte growth factor (HGF) and vascular endothelial growth factor (VEGF) [9].

The injection of single-cell suspensions is a common method for convenient delivery, such as intravenous injection, percutaneous intracoronary injection, and peripheral intravenous injection [10]. However, after injection of the suspension, only a few cells are actively engrafted into the affected tissue. Furthermore, suspension injections cause severe loss, death, and uneven local distribution of cells, which reduces the therapeutic effect of the treatment [11]. Additionally, after prolonged ischemic myocardial infarction, the wall of the left ventricle becomes thinner [12]. Therefore, injecting cell suspension into the infarct zone is difficult.

There is a range of biodegradable materials available for tissue engineering, including polycaprolactone (PCL), polylactic acid (PLA), polyglycolic acid (PGA), and poly(lactic-co-glycolic acid) (PLGA) [13]. PCL has limitations such as a slow degradation rate and low cell adhesion, making it challenging to use as a substrate for culturing MSCs and subsequent transplantation. On the other hand, PLA is more suitable for orthopedic applications due to its prolonged degradation time and strength characteristics. PGA, with its short degradation time, is not suitable for in vitro tissue sheet construction [13–15]. However, PLGA is a biodegradable and biocompatible copolymer, which is widely used as a therapeutic device in drug delivery and tissue engineering applications. And it has been proved for clinical use in humans by the U.S. Food and Drug Administration (FDA) [16, 17]. Furthermore, the rate of degradation and mechanical properties could be controlled by adjusting the ratio of monomers in the combined polymerization of the PLA and PGA without affecting the compatibility and biodegradation [13, 16]. In our previous studies, we have successfully developed 3-dimensional human induced pluripotent stem cell-derived cardiomyocyte (hiPSC-CM) tissue using a PLGA fiber scaffold for the treatment of myocardial infarction in various animal models, ranging from rodent to porcine [18–20]. The PLGA fiber scaffold demonstrated excellent biocompatibility with the cells. Based on these promising results, we have decided to utilize the PLGA fiber scaffold for the construction of organized MSC tissue sheets in present study. In this study, we employed an in vitro method to culture hADSCs on a poly(lactic-co-glycolic acid) (PLGA) fiber scaffold, resulting in the development of a thick and functional hADSC tissue sheet. Then, we evaluated the structure, thickness and condition of tissue sheet by the HE staining and immunofluorescence staining. The paracrine function of the tissue sheet was further evaluated by ELISA assay and Cytokine array. Through the above methods, we found that the hADSC tissue sheet had a thickness of 298.53 ± 18.34 μm and displayed abundant Extracellular (ECM), and considerable cytokine secretion. When utilized for transplantation, we performed echocardiograph and tissue section staining and found that the hADSC tissue sheets effectively improved cardiac function, decreased fibrosis, and enhanced angiogenesis. In short, this approach may offer an efficient and practical method for producing thick MSCs tissue sheets, which could lead to the development of new, clinically effective therapies for treating MI.

## Materials and methods

### Construction of PLGA fiber scaffold

To construct the scaffold, PLGA (75/25; Sigma-Aldrich, St. Louis, MO, USA) was mixed with hexafluoro-2-propanol (HFIP, Wako Pure Chemical Industries, Tokyo, Japan) in a centrifugal tube (1.2 g:3 ml, w/v). The fibers were then synthesized using an automated electrospinning machine (NF-103, MECC, Fukuoka, Japan). The mixed solution was loaded into a 3-mL syringe, to which a needle with a 0.6 mm inner diameter was attached, connecting it to the positive electrode of the high-voltage power supply (10 kV). A layer of aluminum foil was attached to the grounded drum, which was rotated at 1000 rpm to collect the PLGA fiber scaffold. The distance between the needle tip and drum was maintained at 15 cm, and the spinning process was performed for 60 min. Finally, the fiber sheet was transferred to a polydimethylsiloxane (PDMS) frame (1 cm × 1 cm) for subsequent cell seeding. After construction, the fibers were examined using a scanning electron microscope, as previously described [19].

### Culture of hADSCs

The hADSCs used in this study were obtained from PromoCell (C-12977, PromoCell, Heidelberg, Germany), and ethical approval was obtained from the ethics committee of Osaka University. Briefly, cryopreserved P2 (passage 2) cells were obtained and then cultured in MSCs Xeno-Free culture medium (Takara Bio Inc., Shiga, Japan) at 37 °C in a 5% CO_2_ incubator. The culture medium was changed every 2–3 days, and upon reaching 80–90% confluence, the cells were detached using TrypLE Select (Gibco, Waltham, MA, USA) for further expansion. Cells from passages 4 to 7 were used in all experiments.

### Characterization of hADSCs

The flow cytometry (BD FACSCanto II; BD Biosciences, Erembodegem, Belgium) was used for immunophenotyping. The hADSCs were harvested after seven passages, dissociated into single cells, washed with phosphate-buffered saline (PBS), and stained with fluorescence-conjugated antibodies (Table 1) including anti-hCD31-PE, anti-hCD34-PE, anti-hCD45-PE, anti-hCD73-PE, anti-hCD90 (Thy1)-PE, anti-hCD105-PE, anti-HLA-G-PE, and anti-HLA-DR-PE. Mouse IgG1 κ isotype was used as a control for cell stainingThe antibodies were purchased from BioLegend (San Diego, CA, USA). FlowJo software v10.5.3 (BD, Franklin Lakes, NJ, USA) was used to analyze the data.

**Table 1:** The list of antibodies used in this study

Antibody name	Dilution	Company	Catalog #
hCD31	1:1000	Biolegend	303106
hCD34	1:1000	Biolegend	343506
hCD45	1:1000	Biolegend	304008
hCD73	1:1000	Biolegend	344004
hCD90	1:1000	Biolegend	328110
hCD105	1:1000	Biolegend	323206
HLA-DR	1:1000	Biolegend	307606
HLA-G	1:1000	Biolegend	335905
Vimentin [D21H3]	1:100	Cell Signaling	5741S
Collagen Type I	1:100	Sigma-Aldrich	C2456
Collagen Type III	1:100	Abcam	ab7778
Fibronectin	1:100	Abcam	ab2413
Phalloidin	1:100	Sigma-Aldrich	49409
VEGF	1:100	Bioss	bs-0279R
WGA	1:200	Invitrogen	w11261
vWF	1:500	Merck Millipore	AB7356
α-SMA	1:100	Dako	M085101-2
CD90 [F15-42-1]	1:100	Invitrogen	MA5-16671
Ki67 [5D7]	1:100	Abcam	ab156956
Human Nuclei [253-1]	1:50	Merck Millipore	MAB1281

The differentiation potential of hADSCs in adipogenic, osteogenic, and chondrogenic lineages was evaluated using differentiation medium (PromoCell, Heidelberg, Germany) for 2, 2, and 3 weeks, respectively. The confirmation of hADSCs differentiation was done using Oil Red O, Alizarin Red S, and Alcian Blue (Sigma-Aldrich) staining. Samples were examined using a fluorescence microscope (BZ-X800; KEYENCE, Osaka, Japan).

### hADSC tissue sheet formation

hADSCs from the 2nd passage were seeded in a 100 mm dish (Falcon 353,003, Arizona, USA) after thawing. When the cell confluence reached approximately 80%, the hADSCs were collected from the dishes and rinsed with PBS. The hADSCs were then seeded onto a fiber scaffold at a density of 3 × 10^6^ cells/cm^2^. During cell seeding, iMatrix-511 (Matrixome, Osaka, Japan) was added at a concentration of 10 g/mL. The samples were cultured in a humidified atmosphere containing 5% CO_2_ at 37 °C for 3–5 days before transplantation, and the medium was changed every 2 days.

Cryosections of the hADSC tissue sheets were prepared, and the TUNEL assay was performed using the Click-IT TUNEL kit (Alexa Fluor 647) according to the manufacturer’s instructions (Invitrogen; Thermo Fisher Scientific, Waltham, MA, USA).

### Measurement of VEGF and HGF in supernatant

The supernatants were incubated at 37°C, 5% CO_2_, and 95% humidity for 24 h. To evaluate the in vitro function of the hADSC tissue sheet, the supernatant obtained from the tissue sheet on the scaffold and normal cultured hADSCs on a dish (monolayered cells) were analyzed using enzyme-linked immunosorbent assay (ELISA), including vascular endothelial growth factor (VEGF, DVE00), and hepatocyte growth factor (HGF, DHG00B, R&D Systems, Minneapolis, MN, USA, Quantikine ELISA), and was performed according to the manufacturer's instructions. The volume of medium per well in the monolayered hADSCs was the same as that in the hADSC tissue sheets. To eliminate the effects of cell number, we normalized the number of hADSC tissue sheets, enabling comparison of the calculated cytokine levels. Fresh culture medium was used as a control.

### Cytokine array

Cytokine levels in the supernatant were measured using the RayBio C-Series Human Cytokine Antibody Array C5 (AAH-CYT-5, RayBiotech, Norcross, GA, USA), following the manufacturer’s protocols. Briefly, membranes were incubated with blocking buffer for 30 min at room temperature, after which the supernatant was added and incubated at 4 °C overnight. The membranes were then washed 5 times with buffer and incubated with Biotinylated Antibody Cocktail overnight at 4 °C. After washing, membranes were incubated with HRP-Streptavidin Concentrate overnight at 4 °C, washed twice, and placed in detection buffer for 2 min. The signals were detected using the ImageQuant Imaging System (ChemiDoc Touch MP, Osaka, Japan, Bio-Rad). Relative cytokine levels were determined by densitometric analysis using the ImageJ software (NIH, Bethesda, MD, USA).

### Animal model of myocardial infarction (MI) creation and tissue sheet transplantation

The animal experiments were conducted in accordance with the guidelines of Osaka University (01–062-000). Male F344/NJcl-rnu/rnu nude rats (7 weeks old, weight: 175.7 ± 8.7 g; CLEA Japan, Inc., Shizuoka, Japan) were used for all experiments. The rats were observed for seven days before surgical induction of MI.

To create an MI model in 8-week-old rats, the left anterior descending artery (LAD) was permanently ligated via the left intercostal approach 2 mm below the left appendage [21]. Briefly, rats were placed in an induction chamber, and a mixture of medical-grade oxygen and 1.5% isoflurane (Mylan Inc., Canonsburg, PA, USA) vapor was delivered at a flow rate of 3.0 L/min. The concentration of isoflurane was gradually increased to achieve a target anesthetic depth as evidenced by the absence of a response to a gentle toe pinch. Once the desired depth was reached, rats were carefully placed on a respirator during surgery to maintain ventilation. Throughout the experimental period, anesthesia depth was continually monitored and adjusted to maintain a consistent level of anesthesia. The LAD artery was ligated with 6–0 polypropylene suture (Ethicon, Johnson & Johnson, USA) (LAD ligation site, see Additional file 1: Fig. S1). After the surgery, the rats were gradually weaned off isoflurane and allowed to recover from anesthesia in a controlled environment. Two weeks after infarction (week 0), transthoracic echocardiography was performed, and rats with left ventricular (LV) ejection fraction (EF) < 55% were selected as the heart failure model rats.

The rats were randomly divided into three groups: (1) MI only (MI), n = 5 (2) treated with 3 × 10^6^ hADSCs cultured on a PLGA fiber scaffold (hADSC tissue sheet), n = 7 and (3) open-chest operation without LAD ligation (Sham), n = 4. For the hADSC tissue sheet group, the PDMS frame of the PLGA fiber scaffold was cut and removed, and the tissue sheet was placed on the infarcted area of the heart between the visceral and parietal pericardium. In the MI group, no intervention was performed except for the open-chest operation. The rats were sacrificed through an anesthetic overdose (5.0 L/min, 1.5% isoflurane) 4 weeks after surgery, and heart tissues were harvested for further analysis.

### Echocardiography

At baseline (week 0), 2 weeks after MI, and at weeks 1, 2, 3, and 4 after transplantation, the rats were anesthetized with 1.5% isoflurane and kept warm on a heated platform. Transthoracic echocardiographic examinations were then performed on the rats under inhaled isoflurane anesthesia by a blinded technician, using the GE Vivid i system (GE Healthcare, WI, USA) equipped with an 11.5-MHz transducer to observe LV systolic and diastolic movements. Two-dimensional (2D) bright mode (B-mode) and motion mode (M-mode) were obtained from a parasternal long-axis view. The left ventricle internal diameter at end-systole (LVIDs) and left ventricle internal diameter at end-diastole (LVIDd) were determined from M-mode images, using averaged measurements from four cardiac cycles, in accordance with the American Society of Echocardiography guidelines. The left ventricle end-systolic volume (LVESV) and end-diastolic volume (LVEDV) were then calculated using the Teichholz method:

$$\begin{aligned} & {\text{LVESV}} = \frac{{7 \times {\text{LVIDs}}^{3} }}{{2.4 + {\text{LVIDs}}}} \\ & {\text{LVEDV}} = \frac{{7 \times {\text{LVIDd}}^{3} }}{{2.4 + {\text{LVIDd}}}} \\ \end{aligned}$$

Other parameters such as ejection fraction, fractional shortening was calculated:

$$\begin{aligned} & {\text{LVEF}} = 100 \times \frac{{{\text{LVEDV}} - {\text{LVESV}}}}{{{\text{LVEDV}}}} \\ & {\text{LVFS}} = 100 \times \frac{{{\text{LVIDd}} - {\text{LVIDs}}}}{{{\text{LVIDd}}}} \\ \end{aligned}$$

### Immunofluorescence and histological analysis

The hADSC tissue sheets were fixed in 4% paraformaldehyde for 30 min and cryosections were prepared for immunohistology. The sections were incubated with primary antibodies (for detailed information on antibodies, refer to Table 1) overnight at 4 °C, followed by washing with PBS and incubation with respective secondary antibodies at 37 °C for 1 h. After counterstaining with 2-(4-amidinophenyl)-1H-indole-6-carboxamidine (DAPI) or Hoechst 33,342, the sections were analyzed using a fluorescence microscope (BZ-X800; KEYENCE, Osaka, Japan) and a Nikon A1 confocal microscope (Nikon, New York, NY, USA).

After four weeks of transplantation, the rats were euthanized for heart tissue harvesting through echocardiography. The heart was sliced horizontally into five sections, from the base to the apex, for 2,3,5-triphenyltetrazolium chloride (TTC) staining. Samples were then incubated in 2% TTC (Sigma-Aldrich, St. Louis, USA) diluted in PBS (pH 7.4) for 30 min at 37 °C. Following this, the samples were fixed in formalin, transferred to ethanol, and embedded in paraffin. Serial sections, 5 μm in thickness, were prepared to examine normal tissue architecture using hematoxylin and eosin (H&E) staining.

The infarct size was measured using Masson’s trichrome staining, and the fibrosis area was evaluated using picrosirius red staining. The infarct size was calculated as follows:

$$\frac{{{\text{epicardial }}\;{\text{infarct }}\;{\text{ratio}} + {\text{endocardial }}\;{\text{infarct}}\;{\text{ ratio}}}}{2} \times 100$$

The epicardial infarction ratio was calculated by dividing the epicardial infarction length by the epicardial circumference, and the endocardial infarction ratio was calculated in a similar manner. The percentage of fibrotic area was calculated as the average ratio of the fibrotic area to the total LV area:

$${\text{percentage }}\;{\text{of }}\;{\text{fibrotic }}\;{\text{area}} = \frac{{{\text{fibrotic }}\;{\text{area}}}}{{{\text{total}}\;{\text{ LV }}\;{\text{area}}}} \times 100$$

For immunohistology of heart tissue sections, dewaxed paraffin sections were washed in PBS, and antigen retrieval was performed in Target Retrieval Solution (pH = 6; DAKO Japan, Inc, Tokyo, Japan) at 121 °C for 10 min. The sections were stained as described above. Additionally, to quantify the density of capillaries and arterioles, von Willebrand Factor (vWF) and smooth muscle actin (-SMA) were used to stain the endothelial cells and vascular smooth muscle cells, respectively, while cell nuclei were stained blue. The neovessels were represented by orange staining, and orange/green double staining represented arterioles, as previously reported [22, 23].

### Statistical analysis

All quantitative data are presented as mean ± standard deviation (SD) and were analyzed using GraphPad Prism 9.5.0 (GraphPad Software, USA). The difference in normal variates was tested using Student’s *t* test within the two groups. One-way analysis of variance (ANOVA) followed by Tukey’s post-hoc test (variance homogeneity) was used for multiple comparisons. Statistical significance was defined as a *p* value < 0.05, (significance was set at *p < 0.05, **p < 0.01, and ***p < 0.001). Bonferroni and Holm multiple comparisons were performed to determine the significance of specific comparisons in the same group at weeks one, two, three, four, and zero (baseline). Statistical significance was defined as *p* value < 0.05, (significance set at ^**#**^*p* < 0.05, ^**##**^*p* < 0.01).

## Results

### Characterization of hADSCs

**Fig. 1:**
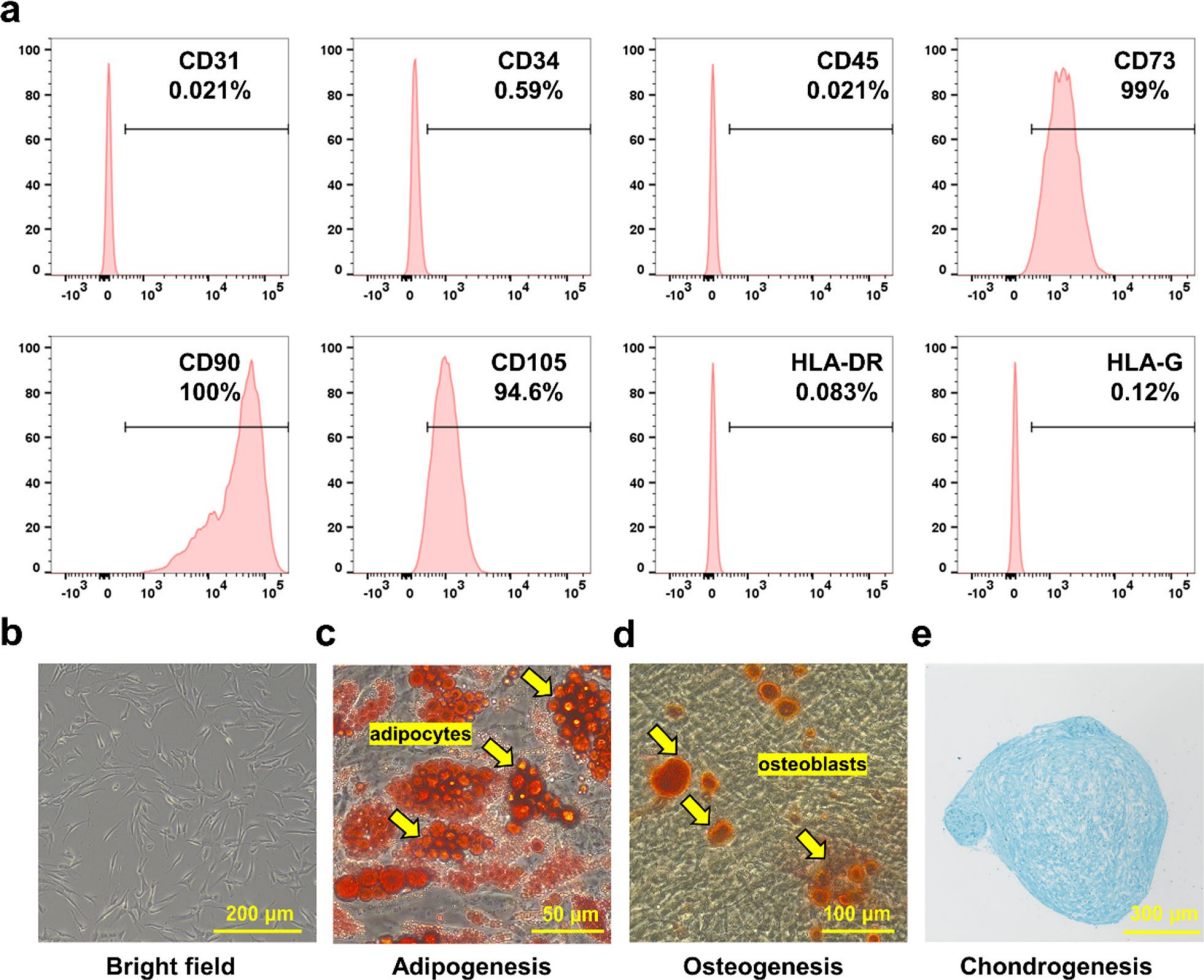
Characterization of human adipose-derived stem cells (hADSCs). **a** Flow cytometry analysis of hADSCs surface markers CD31, CD34, CD45, CD73, CD90, CD105, HLA-DR, and HLA-G. **b** Bright-field microscopic images showing the spindle-like morphology of hADSCs. Scale bar = 200 μm. **c–e** Differentiation potential of hADSCs demonstrated by detection of adipocytes (using Oil Red O staining), osteoblasts (using Alizarin Red staining), and chondrocytes (using Alcian Blue staining). Scale bars = 50 μm, 100 μm, and 300 μm, respectively

To confirm the properties of hADSCs, we conducted flow cytometry analysis to investigate the expression of specific surface markers for mesenchymal stem cells (MSCs). Our findings showed that hADSCs were highly positive for CD73, CD90, and CD105, while significantly negative for CD31, CD34, CD45, HLA-G, and HLA-DR (Fig. 1a). Additionally, the cells exhibited a typical spindle-shaped morphology under bright-field microscopy (Fig. 1b). In vitro differentiation assays were performed after the fourth passage to determine if the hADSCs retained their adipogenic, osteogenic, and chondrogenic differentiation potentials. The cells demonstrated positivity for Oil Red O (adipogenic marker) (Fig. 1c), Alizarin S Red (osteogenic marker) (Fig. 1d), and Alcian Blue (chondrogenic markers) (Fig. 1e). The expression patterns of these surface markers and their differentiation abilities were consistent with the properties of MSCs.

### hADSC tissue sheet construction and evaluation

**Fig. 2:**
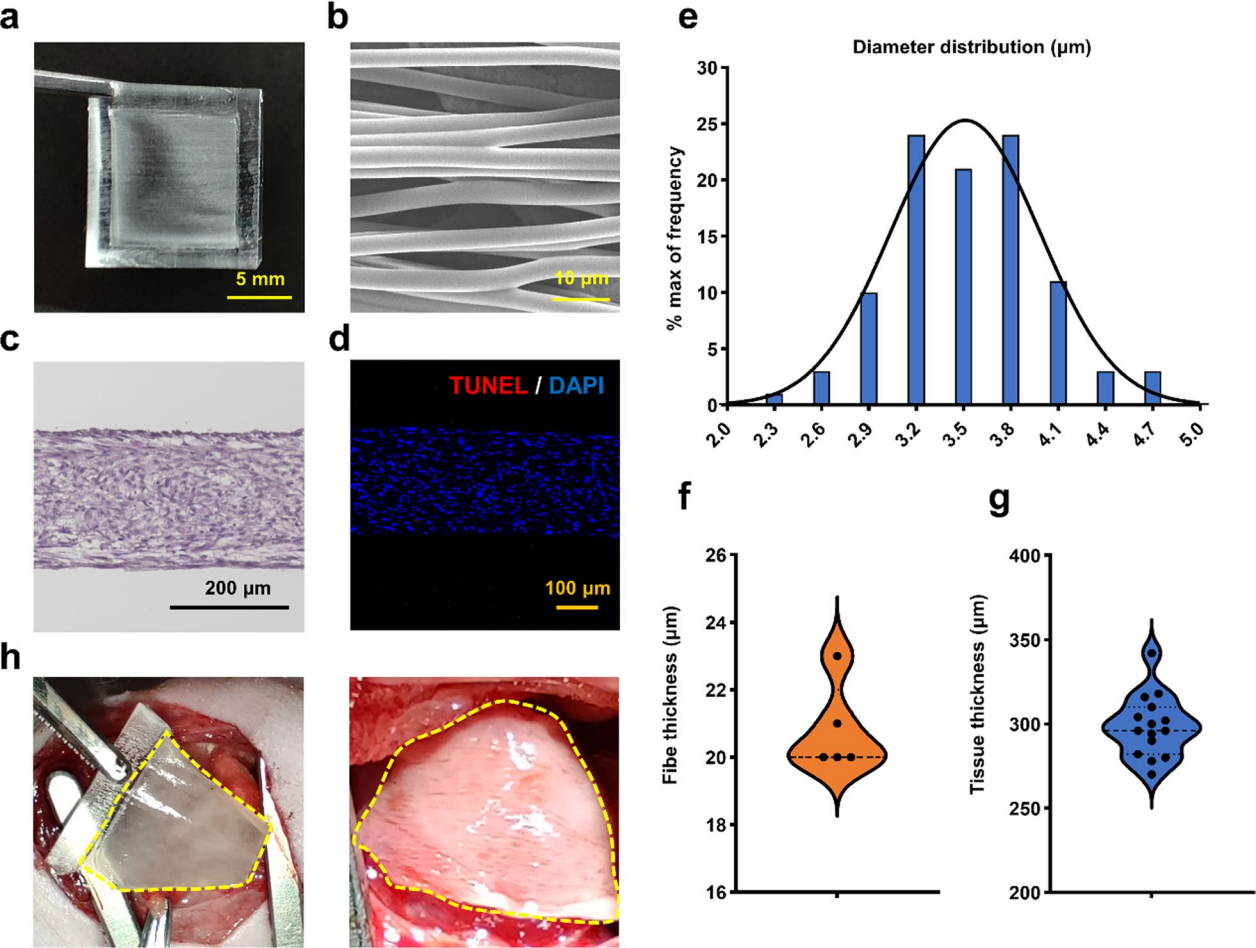
Construction of hADSC tissue sheet. **a** Top view image and **b** Scanning electron microscopy image of PLGA fiber scaffold. Scale bar = 0.5 cm and 10 μm, respectively. **c** Hematoxylin and eosin staining of hADSC tissue sheet (side view). Scale bar = 200 μm. **d** TUNEL staining of hADSC tissue sheet. Scale bar = 100 μm. **e** Quantitative analysis of fiber diameter in PLGA fiber scaffold. **f, g** Quantitative analysis of thickness of PLGA fiber scaffold and hADSC tissue sheet. **h** Operability of hADSC tissue sheet and its transplantation in a MI rat model to cover the ischemic area. MI refers to myocardial infarction, and PLGA stands for poly(lactic-co-glycolic acid)

The PLGA fiber scaffold was utilized as a culture substrate to facilitate tissue formation by hADSCs. The thickness of the PLGA fiber scaffold was 20.8 ± 1.3 μm (Fig. 2a, f), and scanning electron microscopy (SEM) images confirmed the fiber alignment, with the diameter of individual fibers measured at 3.52 ± 0.47 μm (Fig. 2b, e).

The hADSC tissue sheets were constructed using the aforementioned method, and the resulting tissue was evenly distributed on the PLGA fiber scaffold. Hematoxylin and eosin (H&E) staining revealed approximately 30 layers of cell stacking in the vertical direction of the hADSC tissue sheet, with a thickness of 298.53 ± 18.34 μm (Fig. 2c, g). TUNEL labeling demonstrated a low number of apoptotic cells in the hADSC tissue sheets (Fig. 2d, Additional file 1: Fig. S7a–c). And the cell death rate in hADSC tissue sheet was 1.75 ± 0.81% (Additional file 1: Fig. S7d). Moreover, as depicted in Additional file 1: Figs. S4, S5, the cells within the hADSC tissue sheet displayed a characteristic spindle-like morphology and consistently exhibited strong expression of the CD90 surface marker surrounding each nucleus.

In the in vivo transplantation experiments, the hADSC tissue sheet displayed excellent ease of operation and flexibility, attaching effectively to the surface of the infarcted rat heart (Fig. 2h).

**Fig. 3:**
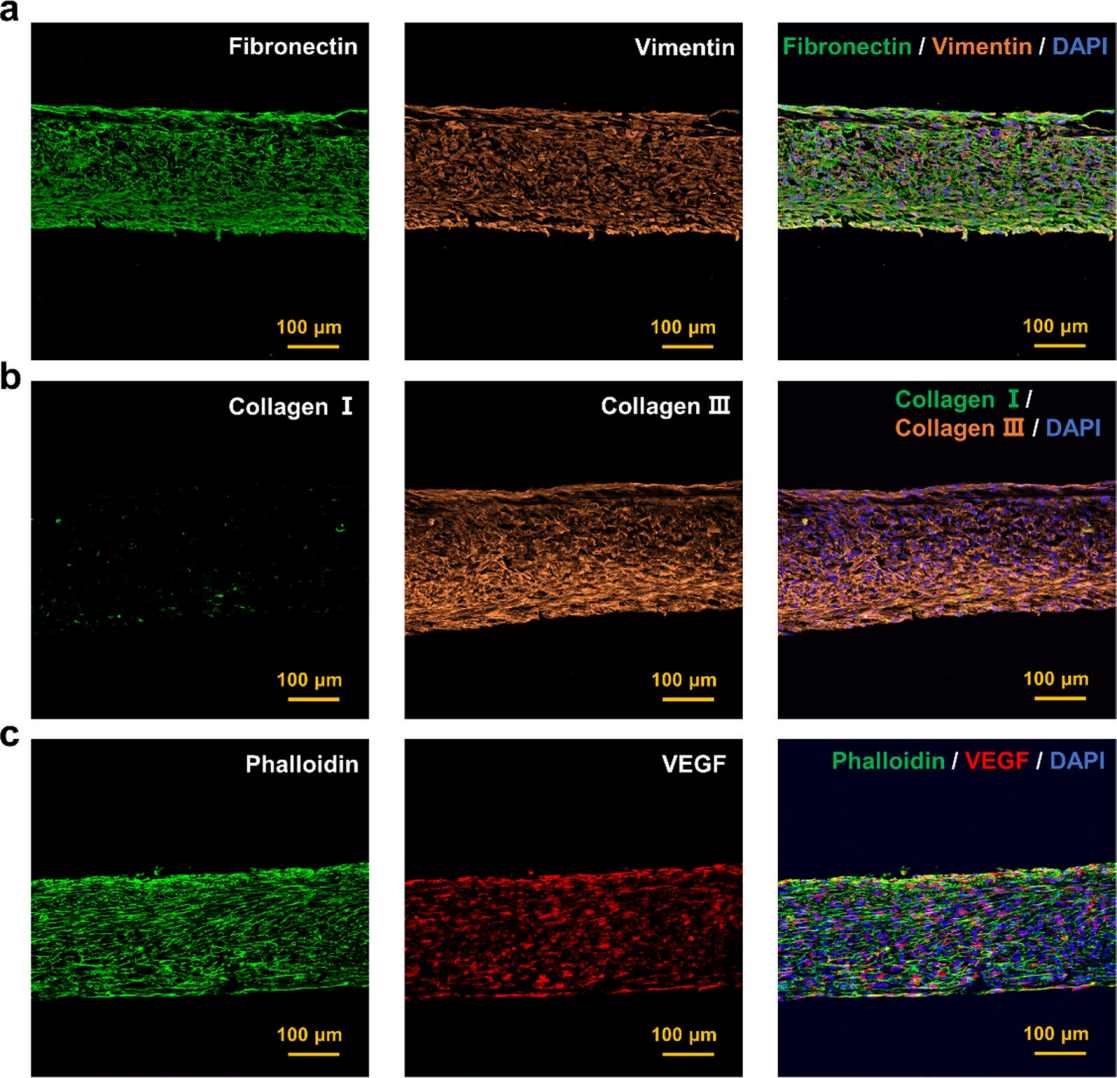
Evaluation of hADSC tissue sheet. **a** Immunohistochemical staining of fibronectin (green) and vimentin (orange) expression in the hADSC tissue sheet. Nuclei were counterstained with DAPI (blue). Scale bar = 100 μm. **b** Immunohistochemical staining of collagen I (green) and collagen III (orange) expression in the hADSC tissue sheet. Nuclei were counterstained with DAPI (blue). Scale bar = 100 μm. **c** Immunohistochemical staining of VEGF (red) expression in the hADSC tissue sheet, with Phalloidin staining for actin filaments (green) and DAPI staining for nuclei (blue). Scale bar = 100 μm

In order to examine the function and paracrine ability of the hADSC tissue sheets, the ECM condition and the abundance of three representative proteins (fibronectin, collagen III, and vimentin) were first identified using cross-sectional immunofluorescence staining (Fig. 3a, b). Additionally, the cross-sectional immunofluorescence staining results showed that the tissue sheet contained abundant VEGF and phalloidin-actin filaments (Fig. 3c).

**Fig. 4:**
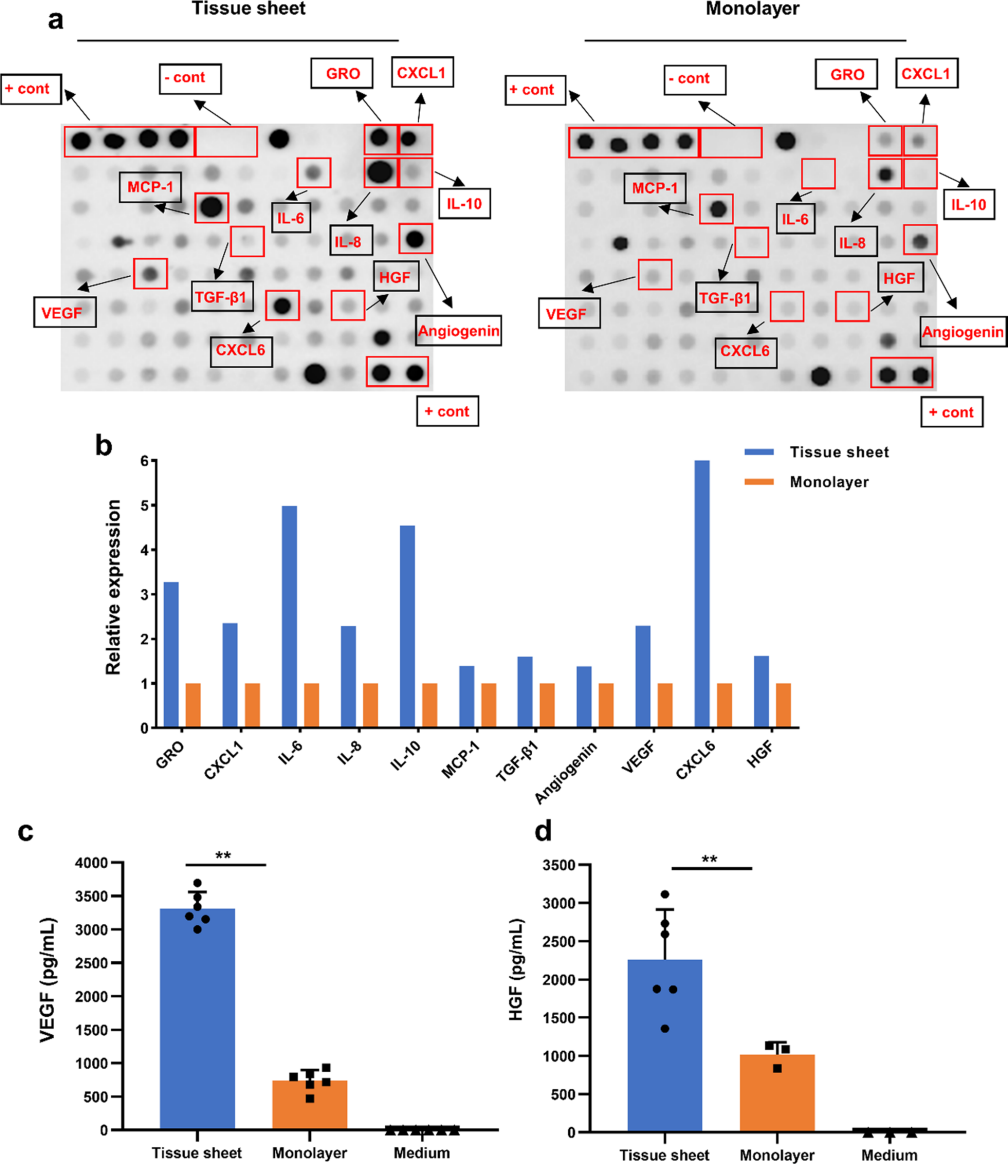
Comparison of cytokine secretion between hADSC tissue sheet and monolayered hADSCs. **a** Representative images of cytokine antibody arrays from the supernatant of hADSC tissue sheet and monolayered hADSCs. Highlighted rectangles indicate the elevated cytokines. **b** Relative secretion of cytokines by hADSC tissue sheet and monolayered hADSCs. **c, d** Comparison of VEGF and HGF secretion in the supernatant of hADSC tissue sheet and monolayered hADSCs. Results are presented as mean ± SD. Significance was determined using the Turkey post hoc test. **p < 0.01. VEGF: vascular endothelial growth factor; HGF: hepatocyte growth factor; SD: standard deviation

To evaluate the paracrine function of the hADSC tissue sheets, a cytokine secretion assay using the Human Cytokine Antibody Array C5 was performed. The cytokines released from the hADSC tissue sheet and hADSC monolayer cells (without tissue sheet construction) are shown in Fig. 4a. The results revealed that the abundance of GRO, CXCL1, IL-6, IL-8, IL-10, MCP-1, TGF-β1, Angiogenin, VEGF, CXCL6, and HGF was significantly greater in the medium from hADSC tissue sheets than in that from hADSC monolayer cells (Fig. 4b). To further investigate the secretion of VEGF and HGF, quantitative analysis was performed using ELISA, as shown in Fig. 4c and d. The concentration of VEGF and HGF in the supernatant of the hADSC tissue sheet was significantly higher than that in monolayered hADSCs (VEGF: 3309.88 ± 249.13 pg/mL vs 737.61 ± 158.62 pg/mL; HGF: 2255.89 ± 659.58 pg/mL vs 1017.90 ± 161.19 pg/mL; *P* < 0.01) (Fig. 4c, d).

Collectively, the PLGA fiber scaffold provided an effective substrate for the formation of well-organized tissue sheets by hADSCs. In addition, the construction of hADSC tissue sheets enhanced both ECM abundance and paracrine secretion.

### hADSC tissue sheet improves cardiac function

**Fig. 5:**
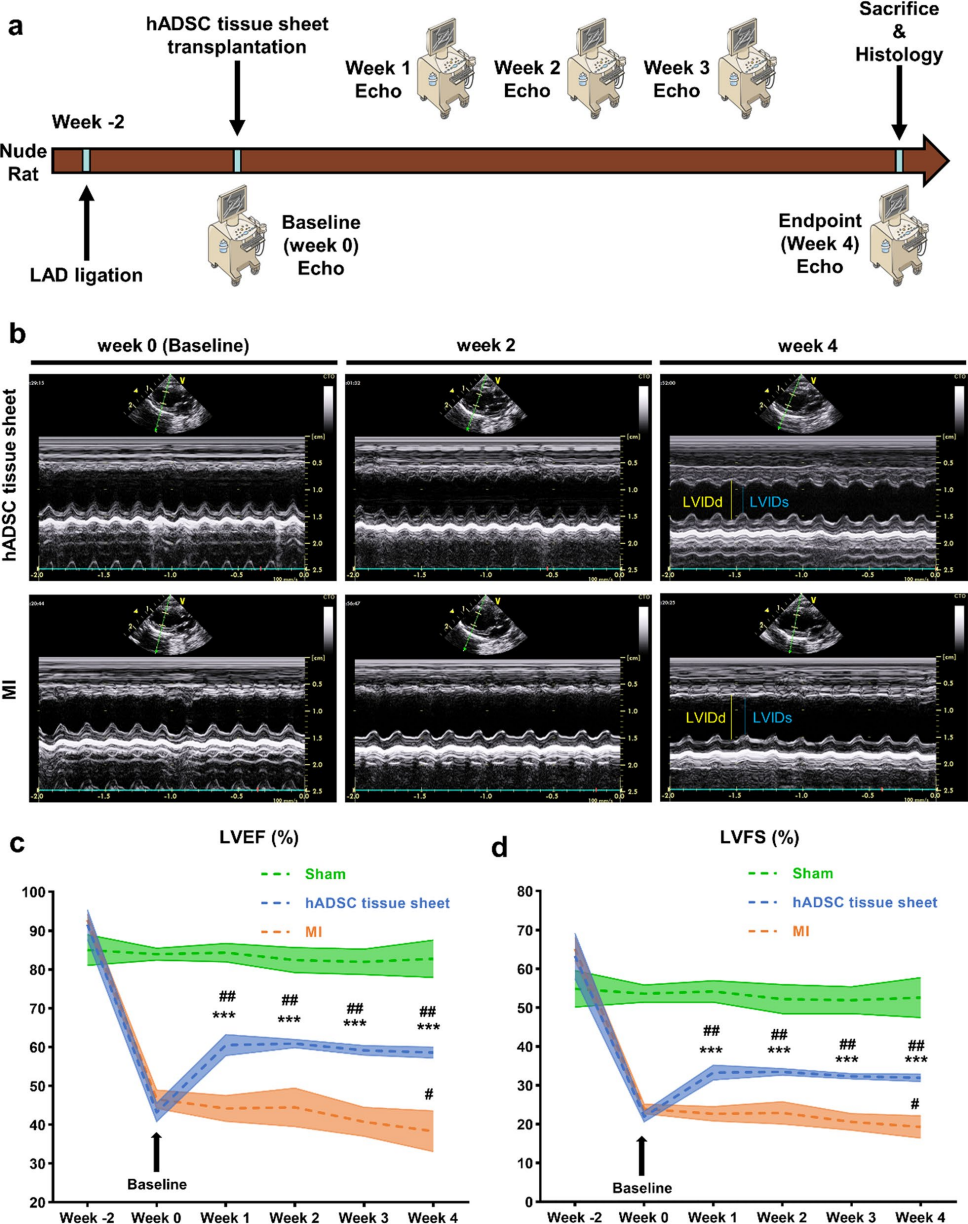
Transplantation of hADSC tissue sheet improves cardiac function. **a** The study protocol of the animal experiment and the evaluation of cardiac function and histological analysis are depicted. **b** Representative long-axis M-mode echocardiography images of the hADSC tissue sheet group and MI group are shown. **c, d** LVEF and LVFS assessed by echocardiography in the Sham group, hADSC tissue sheet group, and MI group before MI, before transplantation (week 0, baseline), and 1, 2, 3, 4 weeks post-MI, respectively (Sham group, n = 4; hADSC tissue sheet group, n = 7; MI group, n = 5). The results are presented as mean ± SD. Significance was determined using Student’s *t* test. ***p < 0.001. Bonferroni and Holm multiple comparison tests were performed to determine the significance of specific comparisons within the same group between week 1, 2, 3, 4 and week 0 (baseline). ^#^*p* < 0.05, ^##^*p* < 0.01. LVEF: left ventricular ejection fraction; LVFS: left ventricular fractional shortening; MI: myocardial infarction; SD: standard deviation

To investigate the cardiac function of rats, we performed echocardiography before and 2 weeks after left anterior descending artery (LAD) ligation and at 1, 2, 3, and 4 weeks after transplantation of the hADSC tissue sheet. The initial left ventricular ejection fraction (LVEF), left ventricular fractional shortening (LVFS), left ventricular internal diameter at end-diastole (LVIDd), and left ventricular internal diameter at end-systole (LVIDs) did not differ significantly among the three groups. LAD ligation successfully induced a significant increase in LVIDd and LVIDs, and a decrease in LVEF and LVFS after 2 weeks in all ischemic models. The baseline LVEF, LVFS, LVIDd, and LVIDs showed no significant differences between the MI and hADSC tissue sheet groups (Fig. 5b–d, Additional file 1: Fig. S2).

As shown in Fig. 5c, rats in the hADSC tissue sheet group exhibited significantly higher LVEF (60.51% ± 2.72%) compared to the MI group (44.17% ± 3.38%) at 1 week (*p* < 0.001), 2 weeks (hADSC tissue sheet 60.90% ± 1.09% versus MI 44.49 ± 4.99%, *p* < 0.001), 3 weeks (hADSC tissue sheet 59.13% ± 1.28% versus MI 40.75 ± 3.71%, *p* < 0.001), and 4 weeks (hADSC tissue sheet 58.62% ± 1.46% versus MI 38.33% ± 5.28%, *p* < 0.001) after transplantation. Similarly, the LVFS values were significantly higher in the hADSC tissue sheet group than in the MI group at 1, 2, 3, and 4 weeks after transplantation, and the trend in the LVFS values was consistent with that of the LVEF values (Fig. 5d).

These findings demonstrate that the hADSC tissue sheet has great potential for improving the cardiac function after myocardial infarction.

### hADSC tissue sheet inhibits cardiac hypertrophy and decreases cardiac fibrosis

**Fig. 6:**
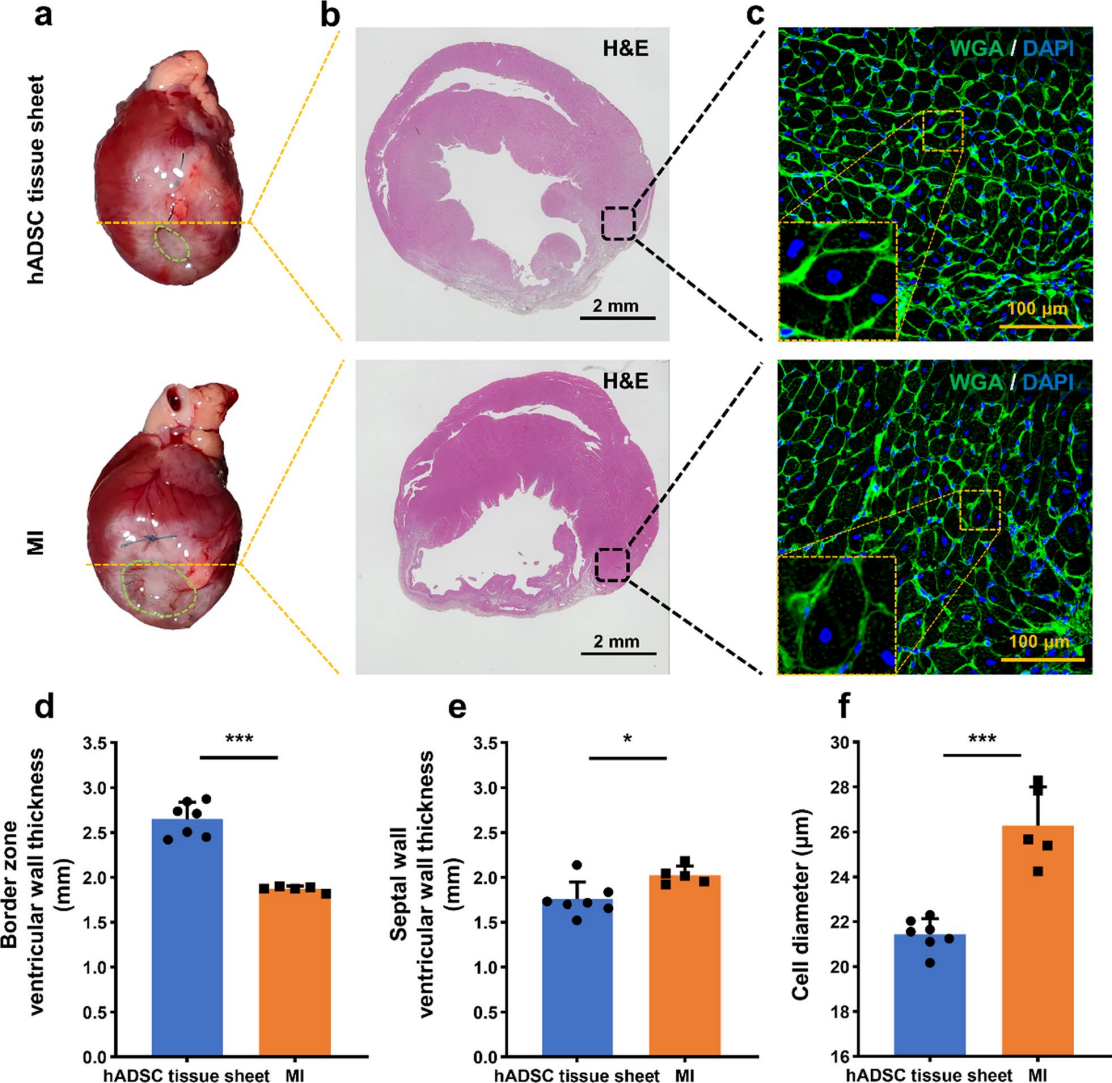
Effect of hADSC tissue sheet on cardiac hypertrophy. **a** Heart morphology of hADSC tissue sheet and MI groups. **b** HE staining of hADSC tissue sheet and MI groups at the papillary muscle level. Scale bar = 2 mm. **c** WGA staining of the myocardium in the border zone with DAPI counterstaining. Scale bar = 100 μm. **d** Quantitative analysis of ventricular wall thickness in the border zone. (e) Quantitative analysis of septal wall thickness. **f** Quantitative analysis of cardiomyocyte cell diameter in the border zone. Results are presented as mean ± SD. Statistical significance was determined using Student’s *t* test. *p < 0.05, ***p < 0.001. MI, myocardial infarction; HE, hematoxylin and eosin; WGA, wheat germ agglutinin; SD, standard deviation

To assess cardiac hypertrophy and fibrosis, we examined the morphological features, H&E staining, and immunofluorescence staining of heart samples obtained 4 weeks after transplantation (Fig. 6a–c). In the border zone, the left ventricular wall thickness was significantly greater in the hADSC tissue sheet group than in the MI group (Fig. 6d). However, in the remote zone (septal wall), the left ventricular wall thickness was higher in the MI group than in the hADSC tissue sheet group (Fig. 6e). Additionally, the cardiomyocyte size (short-axis diameter) in the border zone was significantly smaller in the hADSC tissue sheet group than in the MI group (Fig. 6c, f).

**Fig. 7:**
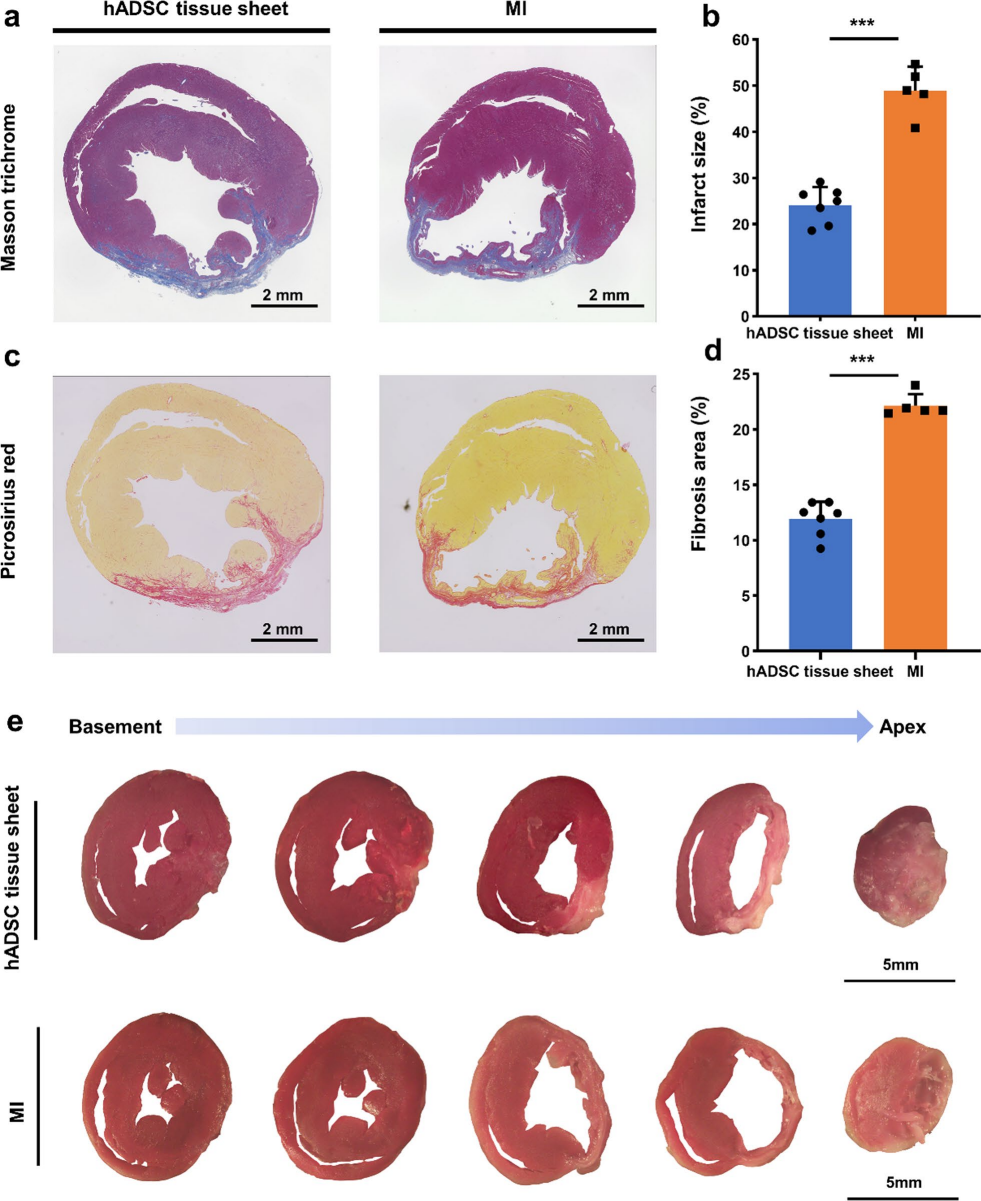
The effect of hADSC tissue sheet on cardiac fibrosis. **a** Masson trichrome staining images of hADSC tissue sheet group and MI group are presented. Scale bar = 2 mm. **b** Infarct size at 4 weeks after transplantation was quantitatively analyzed for hADSC tissue sheet group (n = 7) and MI group (n = 5). **c** Representative Picrosirius red staining images of hADSC tissue sheet group and MI group are displayed. Scale bar = 2 mm. **d** Fibrosis area at 4 weeks after transplantation was quantitatively analyzed for hADSC tissue sheet group (n = 7) and MI group (n = 5). **e** TTC staining from basement to apex of hADSC tissue sheet group and MI group is presented. Scale bar = 5 mm. Results are expressed as mean ± SD, and significance was determined using Student’s *t* test. **p < 0.01, ***p < 0.001. MI, myocardial infarction; TTC, 2,3,5-triphenyltetrazolium chloride; SD, standard deviation

Furthermore, the infarct size and fibrosis area were evaluated by using Masson's trichrome, picrosirius red, and 2,3,5-triphenyltetrazolium chloride (TTC) staining (Fig. 7). The hADSC tissue sheet group showed reduced infarct size compared to the MI group at 4 weeks post-transplantation (Fig. 7a, b). Similar to the infarct size results, the hADSC tissue sheet group demonstrated a smaller fibrotic area than the MI group (Fig. 7c, d). TTC staining also revealed that the MI group exhibited considerable myocardial damage compared to the hADSC tissue sheet group (Fig. 7e), suggesting that the application of the hADSC tissue sheet inhibited the progression of myocardial damage induced by MI.

In summary, these results suggest that the hADSC tissue sheet has greater potential to prevent cardiac fibrosis in the ischemic border zone than in the MI group.

### hADSC tissue sheet promotes angiogenesis in the MI border zone

**Fig. 8:**
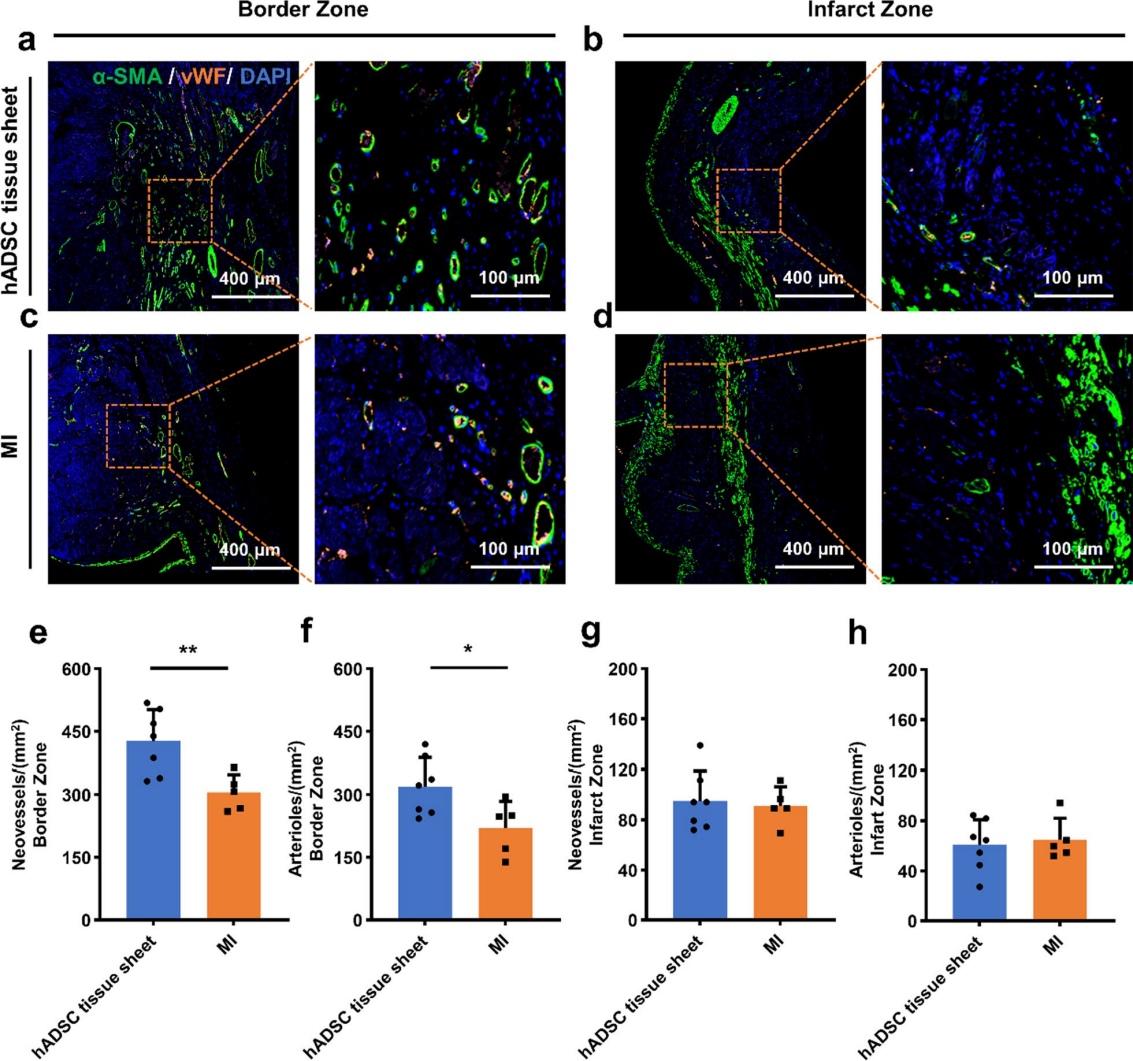
Assessment of angiogenesis at 4 weeks after transplantation. **a–d** Immunostaining of neovessels marker vWF (orange), smooth muscle marker (α-SMA) (green), and nuclei (blue) in the ischemic heart border zone and infarct zone of hADSC tissue sheet group and MI group at 4 weeks after transplantation. Scale bar = 400 μm and 100 μm, respectively. **e–h** Quantitative analysis of vWF- and α-SMA-positive cells/mm^2^ (hADSC tissue sheet group, n = 7; MI group, n = 5). Results are presented as mean ± SD. Significance was determined using Student’s *t* test. *p < 0.05, **p < 0.01. MI, myocardial infarction; vWF, von Willebrand Factor; SD, standard deviation

To investigate the mechanisms underlying the improvements in cardiac function and reduction of fibrosis, we examined the formation of new blood vessels in the infarct and border areas. The hADSC tissue sheet treatment augmented the preservation or formation of neovessels, as shown by immunostaining for vWF, and the arteriolar response, as shown by immunostaining for α-SMA (Fig. 8a–d).

The neovessel density in the border zone was significantly higher in the hADSC tissue sheet group than in the MI group (Fig. 8e). Additionally, the arteriolar density in the border zone was significantly higher in the hADSC tissue sheet group than in the MI group (Fig. 8f). However, in the infarct zone, there were no significant differences in neovessel or arteriolar densities between the two groups (Fig. 8g, h).

These results demonstrate that hADSC tissue sheets have a great potential for promoting angiogenesis in the ischemic heart, particularly in the border zone.

## Discussion

In this study, we developed a thick, functional, and easy-to-use tissue sheet by culturing hADSCs on a PLGA fiber scaffold (Figs. 2, 3, 4). The results showed that the transplantation of hADSC tissue sheets effectively treated ischemic heart failure in rodent. The PLGA fiber scaffold-based hADSCs tissue sheet and the tissue sheet containing 3 × 10^6^ mesenchymal stem cells were transplanted onto the epicardial surface. Following cell transplantation, echocardiographic results indicated a significant improvement in cardiac function (Fig. 5). Histological findings also showed a decrease in the fibrosis area/infarct size, an increase in neovascular density, and a decrease in ventricular remodeling (Figs. 6, 7, 8).

Many studies have shown that autologous and allogeneic sources of MSCs can improve the outcomes of acute and chronic myocardial infarction [5, 24, 25]. The injection of a single ADSC is considered a promising approach to repair damaged myocardium after myocardial infarction, improve LV function and remodeling, and enhance neovascularization through various paracrine cytokines [26]. However, the existing therapeutic efficacy is still limited due to the unfavorable inflammatory microenvironment generated by the ischemic myocardium [27]. Several strategies have been reported to enhance cell survival and function, including the pretreatment of MSCs with growth factors or drugs [28], hypoxic preconditioning [29], genetic manipulation [22], and thermo-responsive dish cell sheet formation [5]. Cardiac tissue engineering to deliver cells to the myocardium via PLGA fiber scaffolds has also been shown to effectively enhance graft survival and function at small and large animal levels [19, 20]. PLGA is a biodegradable and biocompatible copolymer that has been used in FDA-approved therapeutic devices, although it may cause a mild immune response with the degradation [30]. However, the amount of PLGA employed in the construction of our hADSC tissue sheet is relatively small. The thickness of the PLGA fiber scaffold within the tissue sheet is only 20.8 ± 1.3 μm, whereas the overall tissue sheet measures 298.53 ± 18.34 μm in thickness. The immune response caused by this level of PLGA may be mild. Moreover, previous studies showed that PLGA disappeared in vivo in several weeks [31, 32], and in this study, we did not find any residue of PLGA fiber on the rat hearts and sections of hearts in four weeks. Therefore, we consider that the use of this material in therapeutic strategies is clinically safe. Furthermore, we will continue to systematic research the safety of PLGA fiber scaffold in subsequent large animal experiment and preclinical trials.

In previous studies, our group has demonstrated the formation of solid and functional tissue sheets using a PLGA fiber scaffold and human-derived umbilical cord MSCs (hUC-MSCs), which showed better therapeutic effects than the local injection of single cells in diabetic wound healing [23]. In this study, we chose hADSCs due to their easy accessibility in large quantities, potent differentiation properties, and powerful paracrine effects [33]. Additionally, hADSCs have been found to have relatively high levels of HGF, which supports cell survival, suppresses inflammation, and reduces fibrosis [34].

We used hADSCs to construct tissue sheets for cell transplantation, which can retain abundant ECM and produce more paracrine cytokines, thus reducing initial cell loss, leading to early improvement in cardiac function, and decreasing the infarct size and fibrosis area. While previous studies have utilized thermo-responsive dish cell sheet formation to enhance cell retention and improve the cell transplant microenvironment to treat ischemic heart disease [5, 10, 24], tissue produced by this method has limited organization and is difficult to handle during transplantation. Therefore, our one-step technology, that is, tissue construction in only one-step workflow of seeding cells, can obtain highly organized tissue sheets with an extremely simple culturing workflow and is easier to operate during transplant surgery, providing significant advantages in further clinical applications.

The paracrine effect is considered to be the primary mechanism underlying the ability of MSCs to induce cardiac repair rather than differentiate into cardiac cell lineages [35]. In this study, it was found that the hADSC tissue sheet secreted more VEGF and HGF into the supernatant compared to the monolayer cells (Fig. 5). We speculate that during the construction of the tissue sheets, the cells are cultured at high density, resulting in a relatively hypoxic microenvironment [36, 37] that increases the secretion of a series of cytokines, including VEGF and HGF. In addition, our cytokine antibody array results showed altered expression of many cytokines (Fig. 5), which is consistent with previous studies [38, 39]. We believe that the increased secretion of GRO, VEGF, and Angiogenin may be related to angiogenesis in the border zone (Fig. 5), while the increased HGF secretion may be associated with decreased fibrosis [40]. The upregulation of IL-6, IL-10, and TGF-β1 may protect the rat heart through anti-inflammatory mechanisms [40, 41]. However, changes in CXCL1, MCP-1, and CXCL6 expression may be related to other mechanisms in the process of myocardial infarction, which requires further research effort.

Abundant ECM expression is another advantage of our hADSC tissue sheet technology, which allows hADSC tissue sheet to work at a higher density and have more secretion than MSC cell sheet and MSC suspension [5, 42]. As shown in Fig. 4, histological staining revealed abundant ECM expression.

Nonetheless, the fundamental question was whether hADSC tissue sheet treatment could improve cardiac function. The LV long-axis view method for evaluating LVEF and LVFS can reduce human error caused by the selection of a short-axis cross section, thus increasing the accuracy of LV function results. The significant increase in LVEF and LVFS due to the hADSC tissue sheet might be related to better prevention of fibrosis and improved angiogenesis. Our histological results showed that the hADSC tissue sheets decreased fibrosis in the ischemic border zone, which is consistent with the findings for neovessels and arterial angiogenesis. However, the results are limited by the rat model used, and hADSC tissue sheets should be tested in large animal MI models, which will be more clinically relevant in future.

## Conclusion

In conclusion, we have developed thick, functional, and easy-to-operate hADSC tissue sheets using a one-step strategy. The hADSC tissue sheet exhibited a well-organized structure and abundant ECM deposition, and cytokine secretion was enhanced in vitro. In the rat MI model, the hADSC tissue sheet showed high operability, and rats receiving tissue sheet transplantation demonstrated improved functional recovery, reduced fibrosis, and enhanced angiogenesis compared with the MI group. Therefore, hADSC represents a promising treatment option for ischemic MI.

### Supplementary Information

**Additional file 1**. Supplementary data.

## Data Availability

All data generated and/or analyzed in this study are included in this published article.

## Abbreviations

HF: Heart failure
MI: Myocardial infarction
MSC: Mesenchymal stem cell
hADSC: Human adipose-derived mesenchymal stem cell
VEGF: Vascular endothelial growth factor
HGF: Hepatocyte growth factor
PLGA: Poly(lactic-co-glycolic acid)
ECM: Extracellular matrix
LAD: Left anterior descending artery
PDMS: Polydimethylsiloxane
LVEF: Left ventricle ejection fraction
LVFS: Left ventricle fractional shortening
LVIDs: Left ventricle internal diameter at end-systole
LVIDd: Left ventricle internal diameter at end-diastole
LVESV: Left ventricle end-systolic volume
LVEDV: Left ventricle end-diastolic volume
TTC: 2,3,5-Triphenyltetrazolium chloride
H&E: Hematoxylin and eosin
vWF: Von Willebrand Factor
α-SMA: α-Smooth muscle actin
