# Supplementary material for: Development of a thick and functional human adipose-derived stem cell tissue sheet for myocardial infarction repair in rat hearts
Source: Stem Cell Res Ther. 2023 Dec 20;14:380. doi: 10.1186/s13287-023-03560-9 (PMC10734106; doi:10.1186/s13287-023-03560-9)
Supplement: Supplementary file 1 — Additional file 1. Supplementary data. [file 13287_2023_3560_MOESM1_ESM.docx]

**Supplementary material**


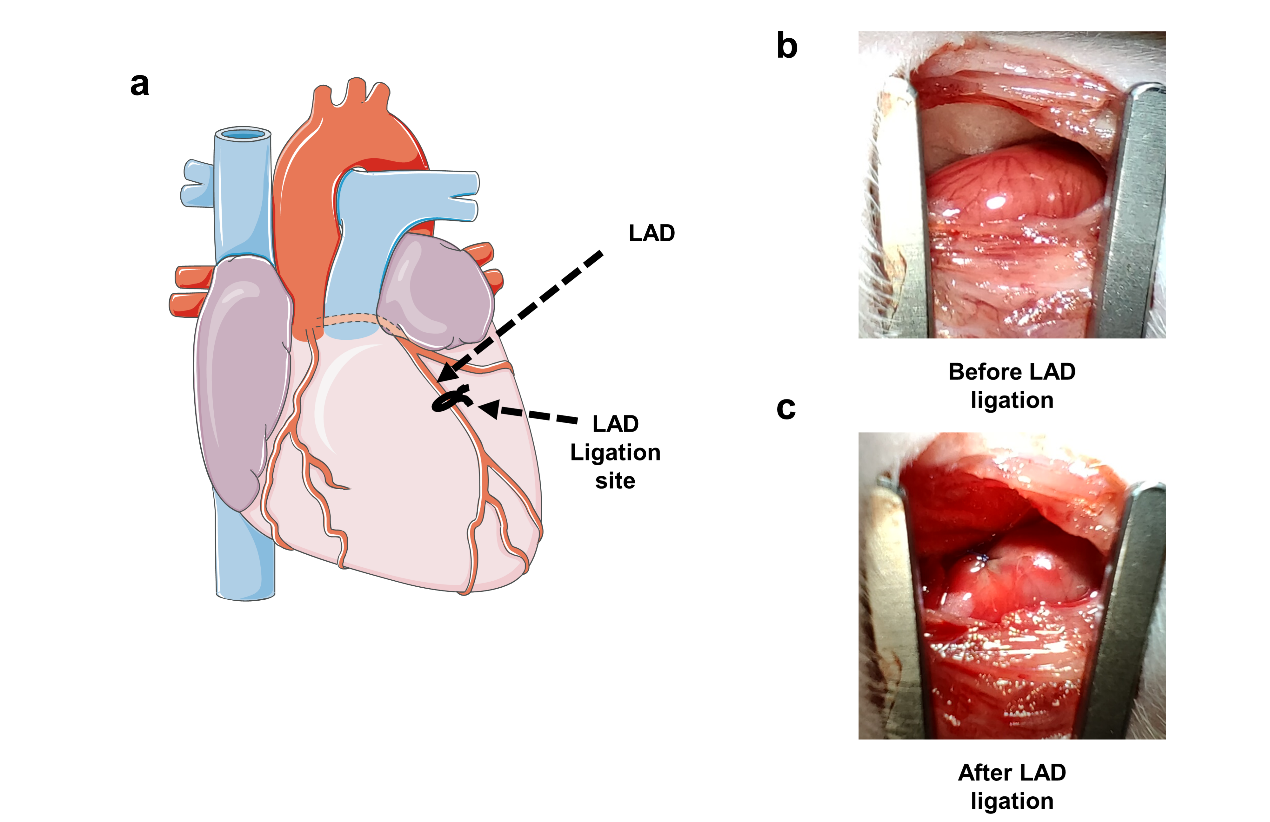


**Supplementary figure 1. (a)** Left anterior descending (LAD) ligation site. **(b-c)** Rat heart before/after LAD ligation.


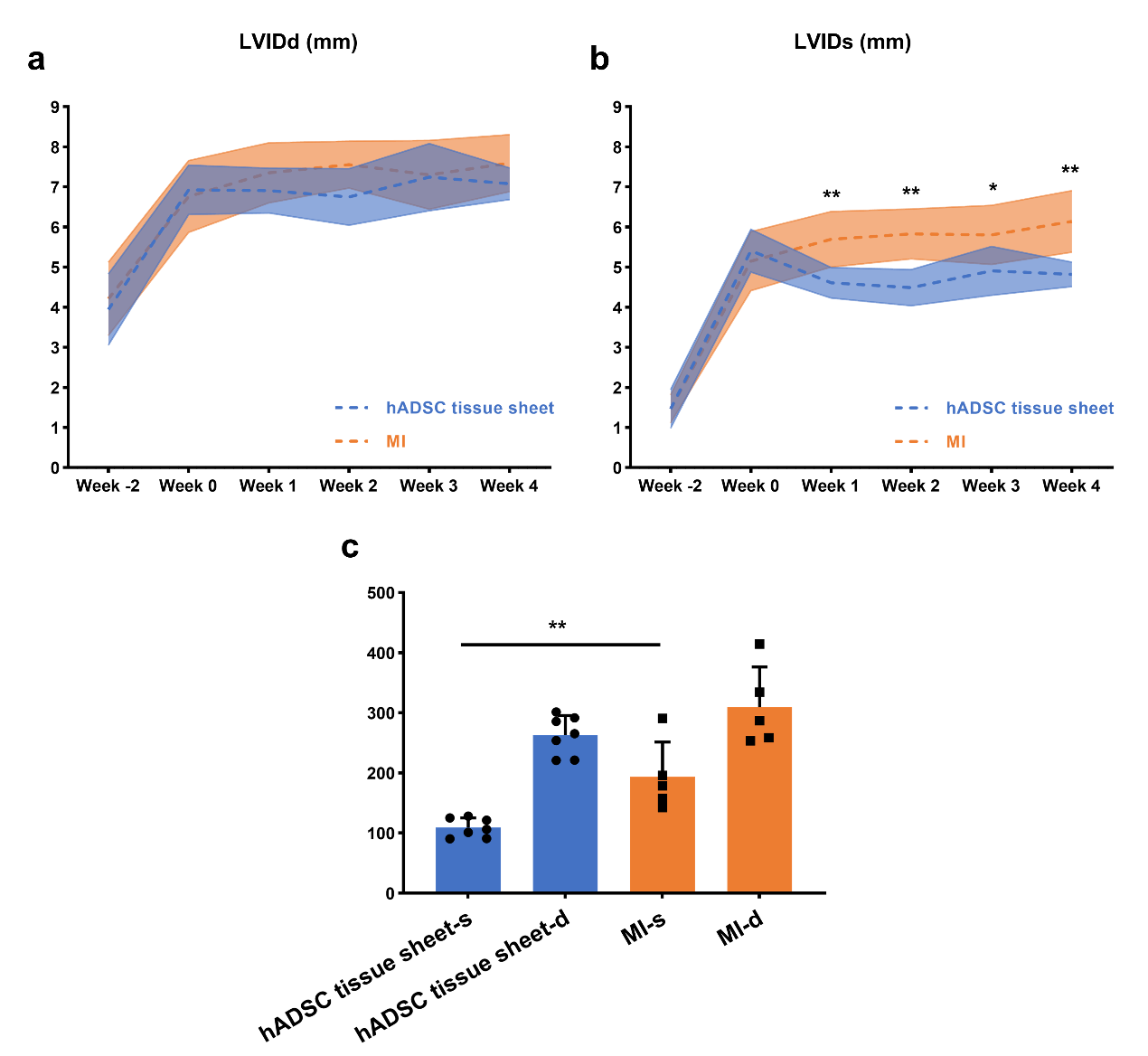


**Supplementary figure 2. (a-b)** Left ventricular internal diameter at end-diastole (LVIDd) and left ventricular internal diameter at end-systole (LVIDs) in the hADSC tissue sheet and MI groups before MI, before transplantation (week 0, baseline), and 1, 2, 3, and 4 weeks post-MI (hADSC tissue sheet group, n = 7; MI group, n = 5). **(c)** left ventricular end end-systolic (-s) and end-diastolic (-d) volumes. Results are presented as the mean ± SD. Significance was determined using Student’s *t* test. * *p* < 0.05, ** *p* < 0.01.


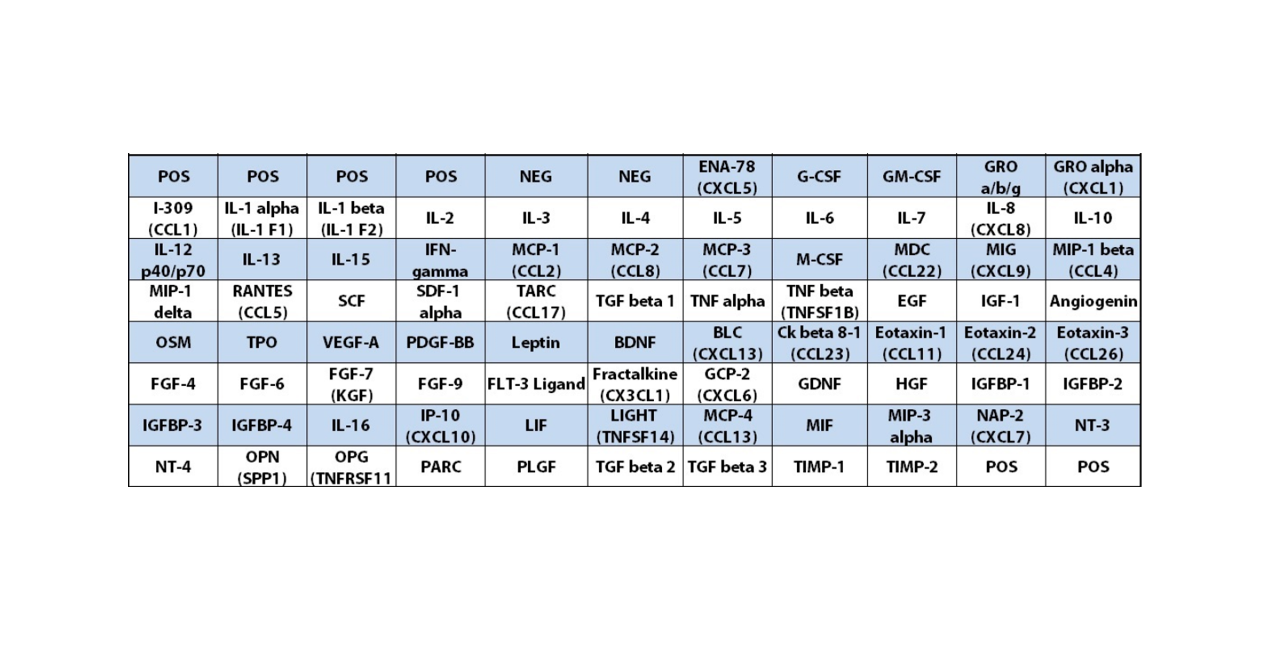


**Supplementary figure 3.** Location and map of cytokines antibodies arranged and spotted on the membrane.


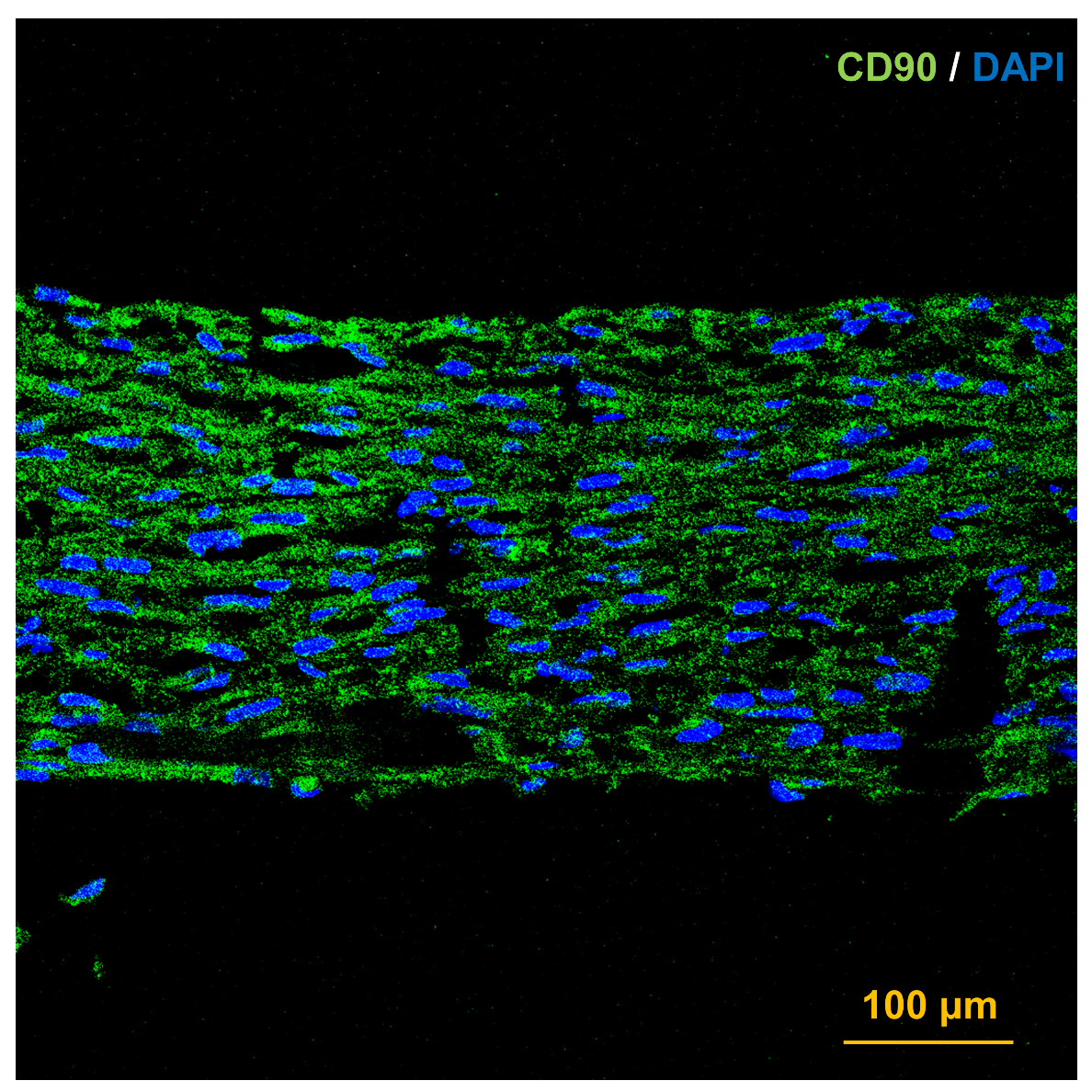


**Supplementary figure 4.** MSCs surface marker (CD90, green) expressed evenly in hADSCs tissue sheet. Nuclei were counterstained with DAPI (blue). Scale bar = 100 μm.


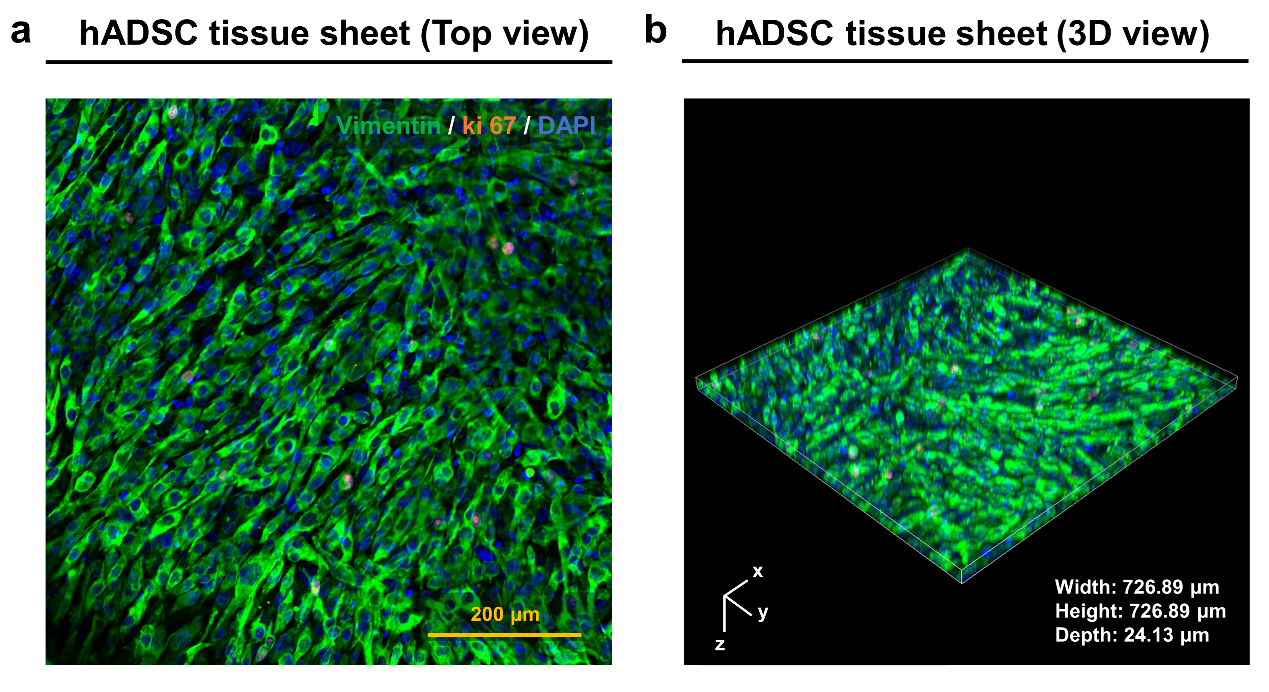


**Supplementary figure 5. (a)** Top view of hADSC tissue sheet. Scale bar = 100 μm. **(b)** 3D view of hADSC tissue sheet.


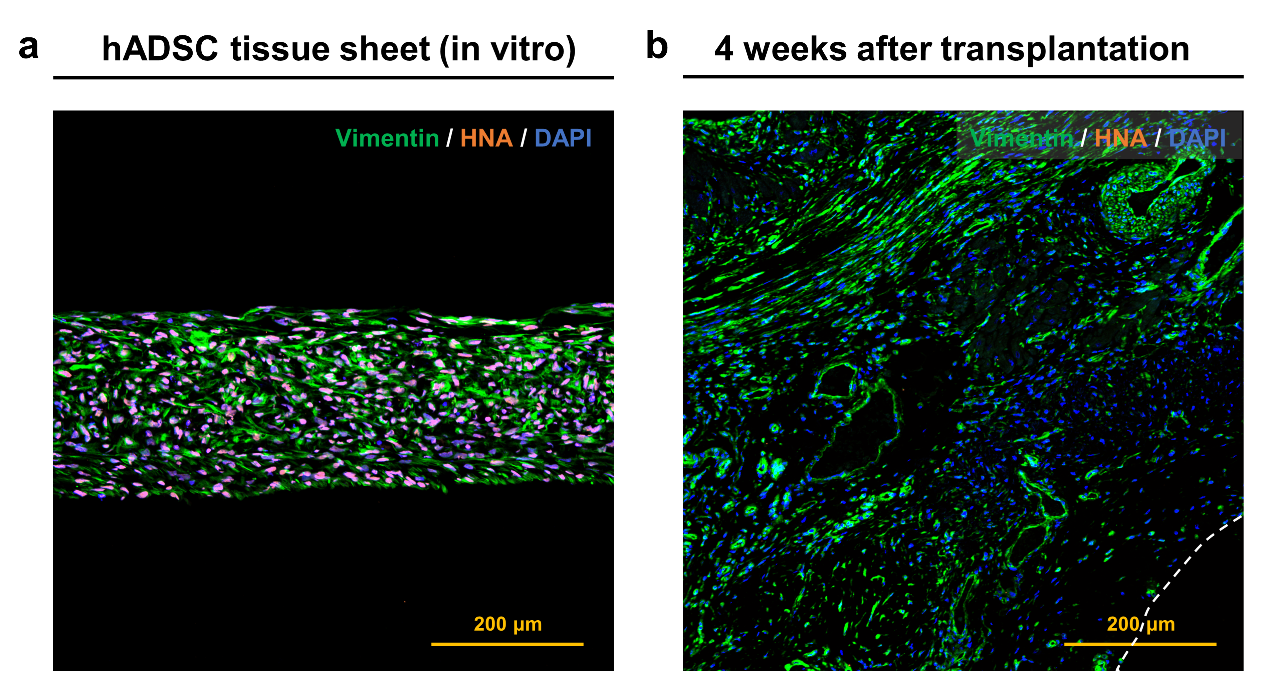


**Supplementary figure 6. (a)** Immunohistochemical staining of vimentin (green) and human nuclei antibody (orange) expression in hADSC tissue sheet. Nuclei were counterstained with DAPI (blue). Scale bar = 200 μm. **(b)** Immunohistochemical staining of vimentin (green) and human nuclei antibody (orange) expression in rat heart tissue section. Nuclei were counterstained with DAPI (blue). Scale bar = 200 μm. The white dotted line indicates the epicardial border.


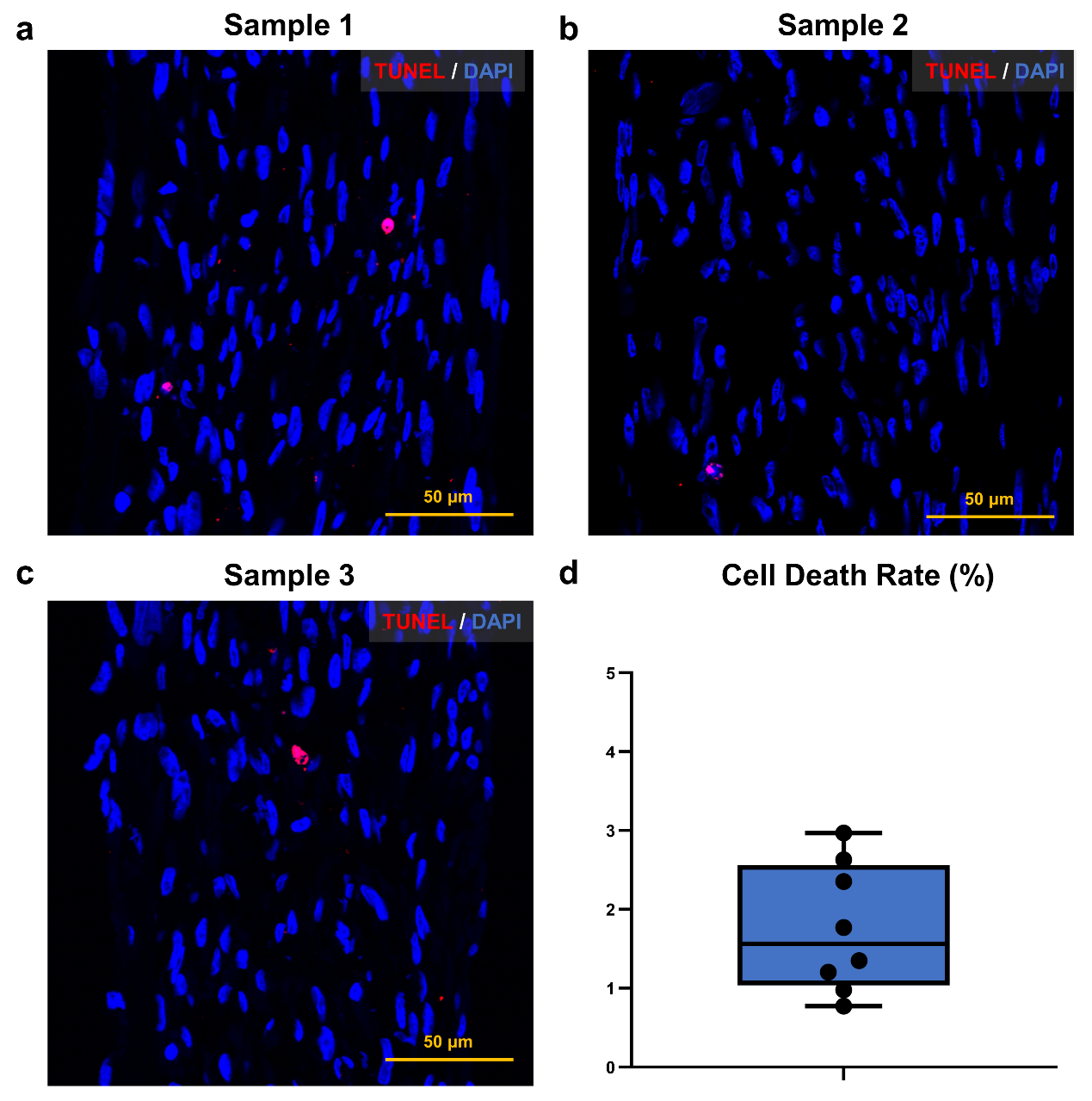


**Supplementary figure 7. (a-c)** TUNEL staining (red) of hADSC tissue sheet (Three representative and independent samples). Nuclei were counterstained with Hoechst (blue). Scale bar = 50 μm. **(d)** Cell death rate of hADSC tissue sheet (n = 8).

**Tables**

**Table 1. The list of antibodies used in this study.**

| **Antibody name** | **Dilution** | **Company** | **Catalog #** |
| --- | --- | --- | --- |
| hCD31 | 1:1000 | Biolegend | 303106 |
| hCD34 | 1:1000 | Biolegend | 343506 |
| hCD45 | 1:1000 | Biolegend | 304008 |
| hCD73 | 1:1000 | Biolegend | 344004 |
| hCD90 | 1:1000 | Biolegend | 328110 |
| hCD105 | 1:1000 | Biolegend | 323206 |
| HLA-DR | 1:1000 | Biolegend | 307606 |
| HLA-G | 1:1000 | Biolegend | 335905 |
| Vimentin [D21H3] | 1:100 | Cell Signaling | 5741S |
| Collagen Type Ⅰ | 1:100 | Sigma-Aldrich | C2456 |
| Collagen Type Ⅲ  Fibronectin | 1:100  1:100 | Abcam  Abcam | ab7778  ab2413 |
| Phalloidin  VEGF  WGA | 1:100  1:100  1:200 | Sigma-Aldrich  Bioss  Invitrogen | 49409  bs-0279R  w11261 |
| vWF | 1:500 | Merk Millipore | AB7356 |
| α-SMA  CD90 [F15-42-1]  Ki67 [5D7]  Human Nuclei [253-1] | 1:100  1:100  1:100  1:50 | Dako  Invitrogen  abcam  Merk Millipore | M085101-2  MA5-16671  ab156956  MAB1281 |
